# Effects of different toothpastes on the prevention of erosion in composite resin and glass ionomer cement enamel and dentin restorations

**DOI:** 10.1590/1678-7757-2020-0493

**Published:** 2020-09-28

**Authors:** Mariana Dias MODA, André Luiz Fraga BRISO, Renata Parpinelli de OLIVEIRA, Núbia Inocencya Pavesi PINI, Diego Felipe Mardegan GONÇALVES, Paulo Henrique dos SANTOS, Ticiane Cestari FAGUNDES

**Affiliations:** 1 Universidade Estadual Paulista Faculdade de Odontologia de Araçatuba Departamento de Odontologia Restauradora AraçatubaSão Paulo Brasil Universidade Estadual Paulista, Faculdade de Odontologia de Araçatuba, Departamento de Odontologia Restauradora, Araçatuba, São Paulo, Brasil.; 2 Centro Universitário UNINGÁ Departamento de Odontologia Restauradora e Prótese MaringáParaná Brasil Centro Universitário UNINGÁ, Departamento de Odontologia Restauradora e Prótese, Maringá, Paraná, Brasil.; 3 Universidade Estadual Paulista Faculdade de Odontologia de Araçatuba Departamento de Materiais Odontológicos e Prótese AraçatubaSão Paulo Brasil Universidade Estadual Paulista, Faculdade de Odontologia de Araçatuba, Departamento de Materiais Odontológicos e Prótese, Araçatuba, São Paulo, Brasil.

**Keywords:** Atomic force microscopy, Composite resins, Glass ionomer cements, Stannous fluoride, Tooth erosion

## Abstract

**Objective:**

This study aimed to evaluate the effects of different toothpastes on the surface wear of enamel, dentin, composite resin (CR), and resin-modified glass ionomer cement (RMGIC), and to perform a topographic analysis of the surfaces, based on representative images generated by atomic force microscopy (AFM) after erosion-abrasion cycles.

**Methodology:**

One hundred and forty bovine incisors were collected and divided into two groups: 72 enamel and 72 dentin blocks (4×4 mm). Half of the specimens were restored with CR (Filtek Z350 XT) and the other half with RMGIC (Fuji II LC). Then, samples were submitted to a demineralization cycle (5 days, 4×2 min/day, 1% citric acid, pH 3.2) and exposed to three different toothpastes (2×15 s/day): without fluoride (WF, n=12), sodium fluoride-based (NaF, n=12), and stannous fluoride-based (SnF_2_, n=12). Surface wear, as well as restoration interfaces wear, were investigated by profilometry of the dental substrates and restorative materials. All representative surfaces underwent AFM analysis. Data were analyzed by two-way analysis of variance and Tukey’s tests (α=0.05).

**Results:**

NaF-based toothpaste caused the greater dentin surface wear (p<0.05). Toothpastes affected only enamel-restoration interfaces. AFM analysis showed precipitate formation in dentinal tubules caused by the use of fluoride toothpastes.

**Conclusions:**

NaF-based toothpastes had no protective effect on enamel adjacent to CR and RMGIC against erosion-abrasion challenges, nor on dentin adjacent to RMGIC material. SnF2-based toothpastes caused more damage to interfaces between enamel and RMGIC.

## Introduction

The number of patients with erosive tooth wear (ETW) has increased during recent years, raising clinical concern.^1^ ETW is the loss of dental substrate caused by physical force, such as toothbrushing, and exposure to acids present in the oral cavity.^1,2^ These acids may either derive from external sources – as fruit juices and soft drinks, which are rich in citric acid, – or from internal sources – as gastroesophageal reflux – and may damage dental substrates over time.^2-4^ETW treatment relies on strategies to improve dental tissues resistance against erosion and, when necessary, on the use of restorative treatments.^5,6^ Composite resins (CR) and resin-modified glass ionomer cements (RMGIC) are often applied in restoration.^7^ However, they are susceptible to erosive acids action, which may decrease their clinical effectiveness and longevity.^8^

Enamel presents a different erosive process than dentin. Whereas on the enamel surface erosion occurs by hydroxyapatite dissolution,^9^ on dentin it begins by peritubular dentin dissolution, exposing the organic matrix, which is rich in collagen fibers and water.^9^ Severe ETW employs the exposure of demineralized organic dentin matrix (DOM), resulting in hypersensitivity and loss of dental tissue in many patients.^9,10^ Typical signs of ETW include development of shallow defects and flattening of the occlusal structures.^11^ Several factors may influence the interaction between acids and dental tissues, leading to ETW , such as: saliva composition and protective capacity, physical force applied during brushing, and toothpaste types and their abrasiveness.^2^

Previous studies investigated anti-erosive toothpastes and their effect on enamel and dentin erosion.^12-16^ Given the protective actions of active compounds on eroded substrates, numerous toothpastes contain active compounds other than sodium fluoride (NaF),^17^ such as hydroxyapatite nanoparticles, potassium nitrate, chitosan, and stannous salts.^12,16,18^ These anti-erosive toothpastes, especially those containing stannous ions, may reduce dentin hypersensitivity by forming a compound that potentially occludes dentinal tubules, decreasing tubular fluid movement induced by external stimuli.^19^ However, some toothpastes that claim to have anti-erosive effect may show high relative dentin abrasivity (RDA).^20^ The literature reaches no consensus on which toothpastes are the most recommended for patients with ETW, and little is known about the surface of restoration interfaces of erosion lesions.

This study aimed to evaluate the effects of different toothpastes on the surface wear of enamel, dentin, CR, and RMGIC after erosion-abrasion cycles. This study hypothesized that (1) toothpastes would present no differences regarding the loss of dental tissues, restorative material surfaces, or restoration interfaces after erosion-abrasion cycles; and (2) analyzed surfaces would present no differences after erosion-abrasion cycles for a single type of evaluated toothpaste.

## Methodology

**Figure 1:**
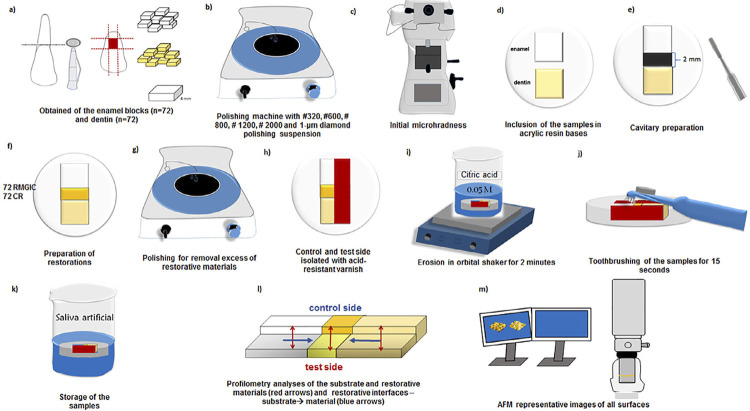
Study flowchart. a,b) Sequence of collection and polishing of enamel and dentin blocks (4×4 mm2). c) Blocks Initial selection by determining microhardness. d) Blocks inclusion, using a metallic matrix e) Cavitary preparation using diamond tip (#1149) f) Restoration with selected restorative material. g) Material excess removal with sandpaper (#1200) h) Application of acid-resistant varnish to create a control side for each specimen. i, j, k) Samples subjected to 5-day erosion and abrasion cycles and storage in remineralizing solution. l,m) Profilometry and AFM analysis performance

This study was approved by the local Animal Ethics Committee (process # 00452-2017). Sample size was determined using the SigmaPlot 14.0 software based in the pilot study with 6 specimens of each group, presenting a minimum difference between the mean (0.55) and standard deviation (0.36) values of profilometry analysis. A significance level of 5% and a power of 80% were adopted, implying a probability of 80% to detect any difference between tested groups. Based on the calculations, a minimum sample size of 12 specimens per group was estimated. Bovine incisors were stored in a 0.1% aqueous solution of thymol for 30 days. Figure 1 shows the study flowchart.

Two specimens (one of enamel and one of dentin) were embedded into acrylic resin using a metal matrix with a 1-mm space for restoration.^6^ A cavity was made on each block mesial side using a diamond bur (#1090, KG Sorensen, Barueri, SP, Brazil) at high-speed rotation. By the end of preparation, the box-shaped cavity had a 2-mm width. Both cavities were filled with the respective restorative material, according to manufacturer’s instructions, and covered with a polyester strip. A glass slide was placed over the strip and a 0.53 kg static load was applied using a heavy glass slab to allow excess material to spill over the top of the cavity margins and ensure it was flat with enamel and dentin surfaces.^6^ Then, the glass slab was removed and the materials were light-cured through the polyester strip and glass slide using a light curing unit at 1000 mW/cm^2^ irradiance (Kavo, Joinville, SC, Brazil). In total, 72 specimens were restored using composite resins (CR; Filtek Z350 XT, 3M ESPE, St. Paul, MN, USA) and photocured for 20 s (Kavo, Joinville, SC, Brazil). The other 72 specimens were restored using resin-modified glass ionomer cements ( RMGIC; Fuji II LC, GC Corporation, Tokyo, Japan), photocured for 40 s, petroleum jelly- coated, and kept under humid conditions at 37°C for 7 days. After storage, samples were polished as previously described to extrude excess material. To create a control surface, a hemiface of each specimen was protected with an acid resistant varnish (Colorama, São Paulo, SP, Brazil).

**Figure 2:**
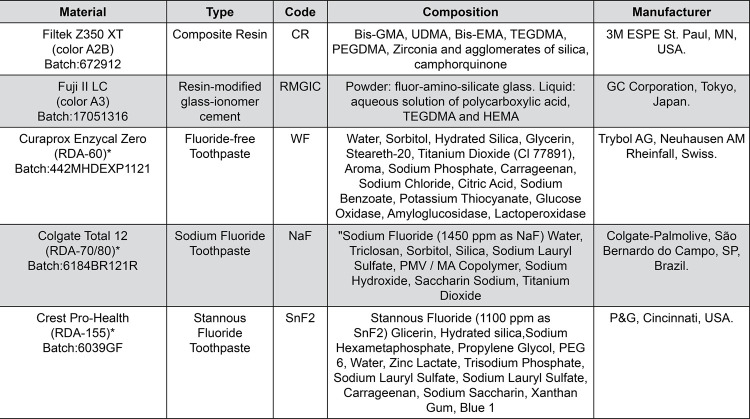
Materials used in this study

Specimens were randomly assigned to 3 experimental groups based on the type of toothpastes used: without fluoride (WF; Curaprox Enzycal Zero, Trybol AG, Neuhausen AM Rheinfall, Swiss), sodium fluoride (NaF)-based (Colgate total 12, Palmolive, Sao Bernardo do Campo, SP, Brazil.), and stannous fluoride (SnF_2_)-based (Crest Pro-Health, P&G, Cincinnati, USA). Figure 2 describes toothpastes and restorative materials specifications.

*RDA values according to manufacturers

### Erosion-abrasion cycling

Specimens were submitted to erosion and abrasion cycles for 5 days. Erosion cycles were performed 4 times daily, and abrasion simulations after the first and last erosion cycles. For erosion, samples were immersed in 250 mL citric acid (PA; Merck, Darmstadt, Germany, pH=3.2), shaken for 2 min using an orbital shaker table (Tecnal TE – 420, Piracicaba, SP, Brazil), and stirred 70 times per minute. After the first and last erosion cycles, for dental abrasion simulations, samples were pipetted with 2 mL toothpaste slurry solution (toothpaste + distilled water in a ratio of 1:3) and brushed using an electric toothbrush on circular motions (Oral-B Plak Control Ultra; Braun, Frankfurt, Germany) with 200 g weight for 15 s. Then, specimens were immersed in the slurry for 2 min.^21^ Erosion cycles were performed with 1 h intervals and, during interim, samples were kept in artificial saliva (1.5 mmol/L^-1^ Ca(NO_3_)_2_.4H_2_0; 0.9 mml/L^-1^ NaH_2_PO_4_.2H_2_O; 150 mmol/L^-1^ KCl, 0.1 mol/L^-1^ Tris buffer; 0.03 ppm F; pH 7.0) at 37ºC.^13^ By the end of the 5-day experimental period, acid resistant varnish was removed and samples were stored at 100% humidity.

### Surface wear analysis

Surface wear was calculated by a mechanical contact profilometer (Surftest SJ 400, Mitutoyo American Corporation, Aurora, IL, USA). At each specimen center, three lines with 2 mm length each (1 mm for the control and 1 mm for the experimental area) were traced^22^ with 0.5 mm intervals. Measurements were also performed on dental surfaces (enamel and dentin), restorative materials (CR and RMGIC), and restoration interfaces with enamel and dentin (Enamel/CR, Enamel/RMGIC, Dentin/CR, Dentin/RMGIC), with 0.5 mm intervals. Scans were interpreted by a specific software (Surftest – SV 2100, Mitutoyo American Corporation, Aurora, IL, USA) and by profilometric evaluation of the regression lines between control and experimental sides. Wear was measured in micrometers and defined as the vertical distance between regression lines on the control surface (previously protected by acid resistant varnish) and the area subjected to erosion-abrasion cycles.

### Atomic force microscopy (AFM)

To visualize different aspects of surface topography, two samples from each group were observed under AFM (Park NX10, Park Systems Corp. Suwon, South Korea). Samples were scanned with a silicon probe tip, under a 0.30 Hz (9 µm/s) scanning rate, and with a 256×256 pixels scanning resolution. A 30×30 µm^2^ three-dimensional image (Gwyddion 2.5, Prague, Czech Republic) was obtained for 6 regions: enamel adjacent to composite resin (ECR); composite resin (CR); dentin adjacent to composite resin (DCR); enamel adjacent to resin-modified glass ionomer cement (ERMGIC); resin-modified glass ionomer cement (RMGIC); and dentin adjacent to resin-modified glass ionomer cement (DRMGIC).

### Statistical analysis

Statistical analyses were performed using Sigma Plot 12.5 software (Systat Software, San Jose, CA, USA). Data were analyzed using the Shapiro-Wilk test (p<0.05). Profilometry data were analyzed using two-way analysis of variance and Tukey’s post-hoc test. Significance level was set at 0.05.

## Results

Table 1 describes the results for dental substrates and restorative materials surface wear. Enamel surfaces (ECR and ERMGIC) showed lower wear than dentin surfaces (DCR and DRMGIC) for all toothpastes analyzed. NaF-based toothpaste caused higher wear on ECR, DCR, and DRMGIC (p=0.015) surfaces than WF and SnF_2_-based toothpastes (p=0.048). However, toothpastes showed no different action on CR, RMGIC, and ERMGIC surfaces (p=0.98; p=0.15; p=0.22, respectively). Both restorative materials showed less surface wear than enamel and dentin, but ERMGIC and RMGIC surfaces showed similar wear with the use of SnF_2_-based toothpaste (p=0.09).

**Table 1:** Mean (SD) of wear (μm) of dental substrates and restorative materials surfaces

	WF	NaF	SnF_**2**_
ECR	4.53 (0.35)^Ab^	7.92 (0.34)^Bb^	5.03 (0.32)^Ab^
CR	0.13 (0.13)^Aa^	0.31 (0.17)^Aa^	0.33 (0.12)^Aa^
DCR	8.58 (0.47)^Ac^	14.53 (0.52)^Bc^	9.88 (0.38)^Ac^
ERMGIC	5.77 (0.24)^Ab^	6.97 (0.52)^Ab^	4.95 (0.38)^Ab^
RMGIC	0.96 (0.24)^Aa^	3.23 (0.36)^Aa^	1.78 (0.21)^Aab^
DRMGIC	10.15 (0.36)^Ac^	13.99 (0.44)^Bc^	9.64 (0.37)^Ac^

Upper case letters compare toothpastes. Lowercase letters compare surfaces.

No compare between specimens restored with CR and RMGIC.

ECR: Enamel adjacent to composite resin; CR:Composite resin; DCR: Dentin adjacent to composite resin; ERMGIC: Enamel adjacent to resin-modified glass ionomer cement; RMGIC: Resin-modified glass ionomer cement; DRMGIC: Dentin adjacent to resin-modified glass ionomer cement.

The negative values of surface wear for restorative materials showed in Table 2 indicates that tissue loss (enamel and dentin) was higher than wear on these surfaces, except for Enamel/RMGIC interface. NaF-based toothpaste caused a higher wear at Enamel/CR interface than SnF_2_-based toothpaste (p=0.003). SnF_2_-based toothpaste caused the greatest wear on Enamel/RMGIC interface, with significant difference compared to other groups (p<0.001). We found no differences in the level of wear for dentin interfaces (p=0.65). By comparing the interfaces between different materials and the same dental substrate, RMGIC showed more surface loss than enamel, and Dentin/RMGIC interfaces showed lower values than Dentin/CR interface (p<0.001).

**Table 2:** Mean (SD) of wear (μm) of restorative interfaces

	WF	NaF	SnF_**2**_
Enamel/CR	-15.10 (0.79)^ABb^	-16.60 (0.89)^Ab^	-11.60 (1.13)^Bb^
Enamel/RMGIC	7.72 (0.45)^Ba^	8.08 (1.04)^Ba^	13.94 (0.59)^Aa^
Dentin/CR	-21.01 (0.75)^Ab^	-22.33 (1.56)^Ab^	-21.95 (1.33)^Ab^
Dentin/RMGIC	-11.74 (0.59)^Aa^	-10.08 (0.58)^Aa^	-11.18 (0.77)^Aa^

Upper case letters compare toothpastes. Lowercase letters compare surfaces.

CR: composite resin; RMGIC: resin-modified glass ionomer cement.

**Figure 3:**
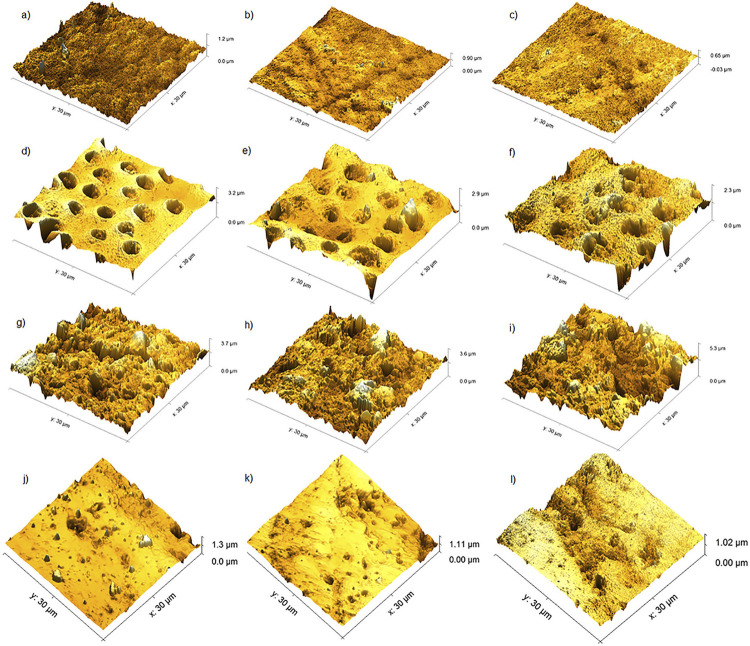
Representative AFM images (256×256 pixels) of enamel, dentin, and restorative materials

Figure 3 shows representative atomic force microscopy (AFM) images. As all eroded surfaces differed from the controls, only eroded-surfaces images are presented, with the aim to illustrate the different effects of different toothpastes on surfaces topography. By comparing toothpastes effects after erosion-abrasion cycle, we observed few alterations on enamel (Figure 3a, 3b, 3c) and CR (Figure 3j, 3k, 3l) surfaces. Samples from the without-fluoride (WF) group presented large dentinal tubules with exposed collagen fibers. Conversely, both NaF and SnF_2_ groups showed partially obliterated dentinal tubules, probably owing to mineral precipitation (Figure 3d, 3e, 3f). RMGIC erosive surfaces presented greater alterations, regardless of the type of toothpaste used (Figure 3g, 3h, 3i).

(a, b, and c): eroded enamel brushed with WF, NaF, and SnF2 toothpastes, respectively; (d, e, and f): eroded dentin brushed with WF, NaF, and SnF2 toothpastes, respectively; (g, h, and i): eroded RMGIC brushed with WF, NaF, and SnF2 toothpastes, respectively; (j, k, and l): Eroded CR brushed with WF, NaF, and SnF2 toothpastes, respectively

## Discussion

Our specimens were prepared based on a method described in a previous study.^6^ Using different restorative materials optimized sample size and enabled an accurate surface wear analysis at the same time. We opted by using citric acid on erosion for being the most common type of acid found in acidic beverages and used in studies involving erosive challenges.^23,24^ Considering that there is no standard protocol for dental erosion cycles, the decision to perform a 5-day erosion protocol with 4 erosion cycles daily was also based on a previous report.^6^ Several variables may affect the results – cycles duration, erosive solution pH, number of cycles performed, and the decision to shake the solution, – hampering a possible correlation of the results with other studies.^23^ We included erosion-abrasion cycles in our study to simulate a more realistic clinical situation.^5^ Some studies evaluated toothpastes available on the market whereas others evaluated manipulated formulations.^12,14-16^ However, our study aimed to evaluate the effects of toothpastes containing different abrasives, according to the relative dentin abrasion (RDA) values reported by manufacturers. The selected toothpastes were manufactured in different countries, but contain active ingredients mentioned in the aforementioned studies.

Profilometry is a quantitative method for evaluating dental tissue loss in relation to a non-treated control area. It is considered the standard method for analyzing *in vitro* and *in situ* tissue loss for erosion or erosion-abrasion simulations.^25,26^ A previous study approached the different types of profilometry (non-contact or contact), differences in the dentinal tissue (wet or dry), and presence or absence of demineralized organic matrix (DOM). The authors concluded that the best method to evaluate dentin was by non-contact profilometry, without DOM.^26^ However, DOM is less thick at shorter demineralization periods, enabling contact profilometry.^26^ Although contact devices may overestimate tissue loss, contact profilometry allows samples to be evaluated within a wet environment, unlike the non-contact type, which uses a light probe.^25^

AFM entails the use of a probe, which provides molecular and atomic level resolution. It evaluates the surface topography of dental tissues, possibly revealing differences between demineralized and remineralized surfaces,^25,27^ as well as the influence of acids, varnishes, or toothpastes.^28^ Although this method allows measurements under ambient conditions (air or liquid), minimizing possible artefacts, scanning a single region takes a long time –completely scanning a region measuring 0.5×0.5 mm takes 60 min.^25^

The surface wear of dental substrates and restoration interfaces involving enamel after erosion-abrasion cycles differed according to the applied toothpaste, rejecting our first null hypothesis. NaF-based toothpaste caused higher levels of wear than WF and SnF_2_-based toothpastes on enamel adjacent to composite resin (ECR), dentin adjacent to composite resin (DCR), and dentin adjacent to resin-modified glass ionomer cement (DRMGIC) surfaces. These results corroborate those reported by a previous study,^14^ in which NaF-based toothpastes caused higher levels of wear than SnF_2_-based toothpastes. Under demineralization conditions, NaF-based toothpastes usually form calcium fluoride (CaF_2_) precipitates on enamel surface, and fluoride ions released in the biofilm increase critical pH for the dissolution of calcium and phosphate in the enamel_._^5,29^ However, in extreme acidic conditions – as in erosion cycles – the formed molecule is unstable, easily soluble, and provides no protection against dissolution.^5^ Fluoride beneficial effects on erosive tooth wear (ETW) rely on other compounds present in toothpastes.^17^ Others studies showed that fluoride and polyvalent metal ions, such as stannous ions, confer a better protection against erosion.^15,16^ The concentration of silica abrasive particles may play a key role in the loss of dental substrates,^15,20^ especially with the use of toothbrush.^5,30^

WF and SnF_2_-based toothpastes caused similar levels of wear on ECR, DCR, and DRMGIC surfaces. While WF toothpaste is a fluoride-free toothpaste, SnF_2_ is considered an anti-erosive agent. Despite the anti-erosive properties of SnF_2_-based toothpaste, silica abrasive particles present on it may decrease its effectiveness due to their ability to bind to stannous ions, decreasing its anti-erosive activity.^15^ By removing the most superficial enamel structure, these particles may also hinder the development of a stannous-rich zone,^15^ which may justify the similar results found for SnF_2_-based and fluoride-free toothpastes. A previous study compared several toothpastes (without fluoride and containing Sn, NaF, and hydroxyapatite) and found toothpastes containing stannous (5.4 μm) to cause the lowest enamel loss, corroborating our results (4.95–5.03 μm).^14^ They also found that, to achieve any beneficial effects, the concentration of abrasive components in these type of toothpastes has to be greater than 10%, or approximately 20% by weight.^15^ Casein-phosphopeptide–amorphous calcium phosphate (FPC-FCA) also promotes better enamel remineralization than NaF. FPC-FCA complex, when able to increase the levels of calcium ions and inorganic phosphate on tooth surface, may be used for inhibiting erosion.^29^ SNF_2_ is theoretically more resistant to erosion for forming a layer on the demineralized enamel and occluding dentinal tubules after an erosive process.^5^ Figure 3d and 3f demonstrate SNF_2_- and NaF-based toothpastes action on dentinal tubule occlusion. Toothpastes did not significantly affect wear levels and topographies of restorative materials (Figure 3g-3l).

Regarding restoration interfaces, SnF_2_-based toothpaste caused higher wear on Enamel/RMGIC interface than WF and NaF-based toothpastes, but we observed no difference between WF and NaF-based toothpastes. This may be explained by the protective effect of stannous ions and the fluoride ions release by the glass ionomer, which could have had a synergistic effect on the eroded enamel surface, decreasing wear.^31^ However, although erosion-abrasion cycles could have been more aggressive to dentin than to enamel surfaces due to histological differences, different toothpastes had no effect on dentin interfaces.^9^

We observed no differences in wear among analyzed surfaces and interfaces for the same toothpaste, rejecting our second null hypothesis. Restorative materials (CR and RMGIC) showed the least wear, followed by enamel and dentin. Another study detected similar wear behavior by profilometry, especially when erosion was followed by abrasion: enamel showed greater wear, followed by glass ionomer, and CR.^32^ Such pattern was observed in yet another study, which applied microhardness to evaluate the percentage of wear after erosion cycle and found both restorative materials to show less wear loss than enamel.^33^ This study showed similar results for enamel adjacent to resin-modified glass ionomer cement (ERMGIC) and resin-modified glass ionomer cement (RMGIC) surface wear for SnF_2_-based toothpaste use, supporting a synergistic effect between RMGIC and SnF_2_ that may protect enamel surface.^31^ For demineralized enamel, the ionomeric material increases the demineralizing solution pH, due to its buffer capacity, and protects the substrate from mineral wear.^31^ Yet, the presence of silica abrasive particles in SnF_2_-based toothpaste, considered anti-erosive, may decrease its protective effect because of the ionic bond formed between silica particles (negative zeta potential) and stannous ion (positive). This reaction may decrease the concentration of available stannous ions, affecting its anti-erosive properties, as described above.^15^ The similarity between ERMGIC and RMGIC wear values is possibly more associated with the ionomeric material effect on the adjacent enamel than with the toothpaste itself.

Enamel prisms could not be precisely distinguished in topographic images generated by AFM (Figure 3) after erosion, possibly because the brushing action had smoothened surface roughness caused by citric acid.^34^ Regarding dentinal surface, NaF and SnF_2_ groups caused a partial obliteration of dentinal tubules, corroborating results found in a previous study.^34^Obliteration could play a role against future acid attacks (Figure 3e and 3f). Composite resin (CR) was less affected by the erosion-abrasion cycles, probably due to its matrix composition (the presence of aromatic rings in its chain, making it more resistant) and the inorganic particles distributed throughout its entire structure, providing a greater resistance to erosion-abrasion challenges.^35^Conversely, the ionomeric material showed a highly altered surface after challenges, with deep cracks and spaces between particles, as well as protruded glass particles from the ionomeric matrix. A study investigating the effect of beverages with different pH values on various resin-based restorative materials (such as Z550 and Fuji II LC) by AFM and scanning electron microscopy analyses observed that glass ionomer presented a damaged surface after erosion-abrasion challenges, while CR presented no significant alterations, regardless of the toothpaste used.^36^

We may point, as a limitation of our study, the presence of DOM in dentin substrate. A previous study showed that profilometry analysis performed in the presence of DOM leads to an underestimation of the actual mineral loss.^26^ However, in our study, the sample did not contain only dentin block from which DOM could be removed, which would have caused dentin to adhere to the restorative material, altering its structure, and compromising sample stability. Besides that, using collagenase to remove the DOM may cause some mineral precipitation, although small, due to the long-term immersion in a calcium-rich solution.^26^ The lack of dental biofilms or a salivary pellicle entailed by the use of artificial saliva in *in vitro* erosion protocols could reduce fluoride retention on surfaces.^6^

The chemical composition of eroded dental substrates in relation to restorative materials still requires further investigation. We suggest future studies to approach the action of these materials on eroded tooth tissue, as well as the chemical changes resulting from erosion/abrasion processes in dental substrates and restorative materials.

## Conclusions

NaF-based toothpastes provided no protective effect against erosion-abrasion challenges on enamel adjacent to CR and RMGIC and on dentin adjacent to RMGIC. SnF_2_-based toothpastes caused higher damage to interfaces between enamel and RMGIC. By analyzing these data, we concluded that anti-erosive therapy should consider toothpastes beneficial effects on treated tissues (enamel or dentin) and restorative materials.
